# Functional Characterization of the Human *Mariner* Transposon *Hsmar2*

**DOI:** 10.1371/journal.pone.0073227

**Published:** 2013-09-11

**Authors:** Estel Gil, Assumpcio Bosch, David Lampe, Jose M. Lizcano, Jose C. Perales, Olivier Danos, Miguel Chillon

**Affiliations:** 1 Department of Biochemistry and Molecular Biology, Edifici H, Center of Animal Biotechnology and Gene Therapy (CBATEG), Universitat Autònoma de Barcelona, Bellaterra, Spain; 2 Department of Biological Sciences, Bayer School of Natural and Environmental Sciences, Duquesne University, Pittsburgh, Pennsylvania, United States of America; 3 Department of Biochemistry and Molecular Biology, Institut de Neurociences, Universitat Autònoma de Barcelona, Bellaterra, Spain; 4 Department of Physiological Sciences II, IDIBELL, University of Barcelona, Campus de Bellvitge, L’Hospitalet de Llobregat, Barcelona, Spain; 5 Institut National de la Sante et de la recherche Medicale U845, Hôpital Necker Enfants Malades, Université Paris Descartes, Paris, France; 6 Institut Català de Recerca i Estudis Avançats (ICREA), Barcelona, Spain; University of Poitiers, France

## Abstract

DNA transposons are mobile elements with the ability to mobilize and transport genetic information between different chromosomal loci. Unfortunately, most transposons copies are currently inactivated, little is known about mariner elements in humans despite their role in the evolution of the human genome, even though the *Hsmar2* transposon is associated to hotspots for homologous recombination involved in human genetic disorders as Charcot–Marie–Tooth, Prader-Willi/Angelman, and Williams syndromes. This manuscript describes the functional characterization of the human HSMAR2 transposase generated from fossil sequences and shows that the native HSMAR2 is active in human cells, but also in bacteria, with an efficiency similar to other mariner elements. We observe that the sub-cellular localization of HSMAR2 is dependent on the host cell type, and is cytotoxic when overexpressed in HeLa cells. Finally, we also demonstrate that the binding of HSMAR2 to its own ITRs is specific, and that the excision reaction leaves non-canonical footprints both in bacteria and eukaryotic cells.

## Introduction

Transposons are small mobile genetic elements widely distributed in the genomes of bacteria, plants, invertebrates and vertebrates, that have the ability to migrate and carry genetic material between chromosomal loci [1]. Transposons have played an important role in the evolution of genes, including humans, where at least 50 genes derive from transposable elements [2,3]. In the human genome, DNA transposons make up to 3% of the genome [2], with nine of the ten eukaryotic superfamílies present, and ranging from hundreds to several thousand copies per family [4], specially the Tc1/*mariner* superfamily which accounts for one forth of all the copies.

Transposons of the Tc1/*mariner* superfamily are widely distributed in both invertebrate and vertebrate species. However, due to functional inactivation by mutations accumulated during evolution no DNA transposon is active in mammals and are very few in vertebrates [5]. Two *mariner* transposons are present in the human genome, *Hsmar1* and *Hsmar2*, and are associated with the cecropia and *irritans* subfamilies respectively. Oosumi and collaborators discovered *Hsmar2* within an inverted repeat structure initially named Humar1 [6]. By increasing the number of sequences available in public gene databases, a consensus sequence *Hsmar2* was built using 16 sequences selected from the 1000 copies already described in the genome [7]. It has been noted that copies of individual *Hsmar2* are highly mutated with respect to the consensus sequence, with numerous indels as well as stop codons in the transposase pseudogene, leaving intact only 3.7% of the hypermutable CpG dinucleotides [7]. The low frequency of CpG suggests that *Hsmar2* evolved in the genome of vertebrates, mutating into CA, TG or derivatives. This pattern of molecular evolution fits the current model of neutral evolution for the *mariner* transposons in the human genome starting in a common ancestor of the initial suborders of primates [8]. Although copies of *Hsmar2* seem to be the remnants of an inactive functional mariner element of the primate lineage, the analysis of hotspot for homologous recombination in the human genome has revealed the presence of *Hsmar2* copies near the hotspot for homologous recombination in, the PWS/AS region (15q13), the WAS region (7q11), and the CMT1A region (17p12), where large deletions, or repeated sequences are involved in the molecular mechanism of genetic disorders as Prader-Willi and Angelman syndromes (PWS/AS) [9], Williams syndrome (WAS) [10], and Charcot–Marie–Tooth [11] respectively. Thus, studies of homologous recombination at the Charcot–Marie–Tooth disease type 1A suggest that the presence of an *Hsmar2* element within two flanking 24-kb repeats (CMT1AREP) may stimulate unequal crossing-over events between misaligned CMT1A-REP elements [11,12]. These unequal crossing-over events can be resolved as either a 1.5-Mb duplication resulting in Charcot–Marie–Tooth disease or a 1.5-Mb deletion resulting in hereditary neuropathy with liability to pressure palsies [13,14]. Moreover, it has been described that of the 109 copies of *Hsmar2* identified by PRINS [15], about 50% of them were in known fragile sites, suggesting that there may be a potential correlation between the localization of *Hsmar2* copies and fragile sites in the human genome.

To study the function and activity of DNA transposons, and considering the absence of any endogenous active copy in the genomes of mammals, several research groups have reactivated inactive Tc1/*mariner* transposons using approaches based on phylogenetic comparisons. For example, the human *mariner* transposon *Hsmar1*, inserted into the SETMAR gene by an event of "molecular domestication'' has been revived using a sequence derived from ancient inactive copies [16]. For Tc1 transposons, both Sleeping Beauty (SB) [17] and the *Frog Prince* elements [18] have been reconstructed from inactive elements of a salmonid genome and the frog *Rana* *pipiens* genome, respectively. Due to their ability to move around the genome, transposons have great potential as genetic tools, and thus most of these elements have been mutated to hyperactivate their transposase activity in order to apply them in genetic strategies such as insertional mutagenesis studies, transpositional transgenesis, or gene therapy strategies in pre-clinical models [19,20].

In this study, we have characterized an active human HSMAR2 transposase generated from inactive Tc1/mariner elements found in the human genome. We show that the overexpression of HSMAR2 is toxic, but this effect is lost when using a negative self-regulated expression cassette. We have also characterized its activity and analyzed each of the successive steps of the transposition process including binding, excision and transposition.

## Materials and Methods

### Reassembling of the *Hsmar2* transposon

More than 400 copies of *Hsmar2* present in the database GenEmbl were initially selected using the ITR (Inverted Terminal Repeats) sequences as probes. After eliminating repeated copies and short sequences with less than 600 nucleotides of homology, 71 sequences with at least one ITR were finally chosen. The mínimum and maximum level of sequence depth was 16 and 41 respectively, and the sequence obtained (*Hsmar2*) was based on majority rule for the alignment. CG dinucleotides were reintroduced in TG or CA dinucleotides with intermediate frequencies because they likely derive from changes in the hipermutable CG dinucleotide during the evolutionary process.

### Cell culture, transfection and adenovirus production

HeLa (ATCC, CCL-2), and HEK-293 (ATCC, CRL-1573) are cell lines of human origin; and C2C12 (ATCC, CRL-1772), S16 (ATCC, CRL-2941), COS-7 (ATCC, CRL-1651) and DKZeo [21] are of murine, rat, simian and canine origin respectively. All cell lines were cultured in DMEM + 10% (v/v) FBS, at 37^°^C and 5% CO_2_. Transfection were performed in 60-70% confluent cultures with DMEM + 1% (v/v) FBS, using 6 µg of DNA complexed with PEI-25 KDa (Aldrich) per 10^6^ cells, as previously described [22]. Neomycin and Zeocin selection was performed using 700 µg/mL of Neomycin or 100 µg/mL of Zeocin. Briefly, media was replaced every three days until individual clones were observed (usually in two weeks). Clones were selected, grown individually and subsequently passaged until 20x10^6^ cells per clone were obtained. Adenovirus vectors were generated, amplified and purified as described before [22].

### Analysis of Hsmar2 expression by Western blot

AbHsmar2-1001, a rabbit polyclonal (protein-A purified) generated in this work was used at a dilution of 1/5000; polyclonal anti-GFP antibody (Invitrogen, A6455) at a dilution of 1/3000; polyclonal antibody anti-actin (Sigma, A2066) at 1/1000; and secondary polyclonal antibody rabbit HRP-anti-Ig (Dako-Cytomation, P0399) at 1/5000. Protein extracts (4-12 µg per sample) were loaded onto denaturing acrylamide gels and further electrotransferred to PDVF membranes (Amersham). Primary antibodies were incubated in the presence of 5% (w/v) skim milk.

### Immunoprecipitation

HeLa cells (2x10^8^ cells) were transfected with 70 µg of pGFP-HSMAR2. Forty-eight hours later, proteins were extracted. One microgram of antibody AbHsmar2-1001 was incubated with 300-500 g of protein extracts, for 2 hours at 4^°^C with gentle agitation. Immunoprecipitation with protein G sepharose (Sigma) was performed following manufacture’s instructions.

### Flow cytometry

HEK293 or HeLa cells (1x10^6^ cells) were transfected in triplicate with increasing amounts of peGFP-HSMAR2 or pKS-RSV/GFP. Irrelevant plasmid p123T (MoBiTec) was added to achieve 2.5 µg (HEK293) and 10 µg (HeLa) of total DNA per condition. At 24, 48, 72 hours cells were harvested and resuspended in 200 µl PBS1X and propidium iodide (0.4 µg) for 15 minutes at 4 ^°^C and further analyzed with Flux FACSCalibur (BD) and Cellquest Software.

### Electrophoretic Mobility Shift Assays (EMSA)

EMSA was performed using biotin-labeled oligonucleotides from the 5’-ITR. Oligonucleotides sequences are: TR_Dir (5’-ttaataaaTACGAGGGGTCTTCAAAAAGTTCATGGAAAATGtatatattaa-3’) and TR_Rev (5’-ttaatatataCATTTTCCATGAACTTTTTGAAGACCCCTCGTAtttattaa-3’). DNA-binding reaction mixtures (20 µl) contained 1nM of labeled duplex substrate, 1µg poly (dI-dC), 3µg nuclear extract, in 10 mM Tris-HCl [pH 8.0], 50 mM KCl, 5 mM MgCl_2_, 1 mM DTT, 2.5% (v/v) glycerol, 0.05% (v/v) NP-40, 0.3 mg/ml BSA, 1 mM EDTA. After 30 min incubation at 37 ^°^C, reactions were loaded onto a non-denaturing 5% acrylamide gel. Biotin-labeled DNA was visualized with Chemiluminiscent nucleic acid detection kit (Pierce) following the manufacturer’s instructions. Signals were quantify by densitometry by the Syngene Imaging system (Syngene).

### PCR analysis of the transposition process

HeLa cells (1x106) were transfected with 6 g of the self-regulated pCA plasmid. After 72 hours of transfection, DNA was extracted, digested by restriction enzymes cutting within the transposon, PCR amplified and sequenced. PCR primers were DIR172 (5’-GTTAGCTCACTCATTAGGCACC-3’) and REV165 (5’-ATGTGCTGCAAGGCGATTAAG-3’). Nested PCR primers were DIR123 (5’-CGTATGTTGTGTGGAATTGTG-3’) and REV86 (5’-TAATACGACTCACTATAGGGC-3’). For nested PCR, 1 µl of a dilution 1/100 from first PCR was amplified in 50 µl, run in agaraose gel 1%, DNA bands purified by Geneclean (Qbiogene), sequenced at the Servei de Seqüenciació (Universitat Autonoma, Barcelona) and BLAST analyzed. For inverted PCR, pOX38 plasmid was extracted and recircularized with T4 DNA ligase, further digested with Exonuclease I of *E. coli* (NEB) and Lambda exonuclease (NEB) and purified. Inverted PCR was performed using 250 ng of DNA in 50 µl of volume. Primers were iPCR-I3R (5’-AAGCAGGCATCGCCATGGGTCA-3’) and IPCR-I4D (5’-AGCGCGGGGATCTCATGCTG-3’). Primers for following nested PCR were N3R (5’-CCTGATGCTCTTCGTCCAGAT-3’) and N4D (5’-GGAATAGGAACTAAGGAGGA-3’).

## Results

### Reassembling of the human mariner transposase HSMAR2

**Figure 1:**
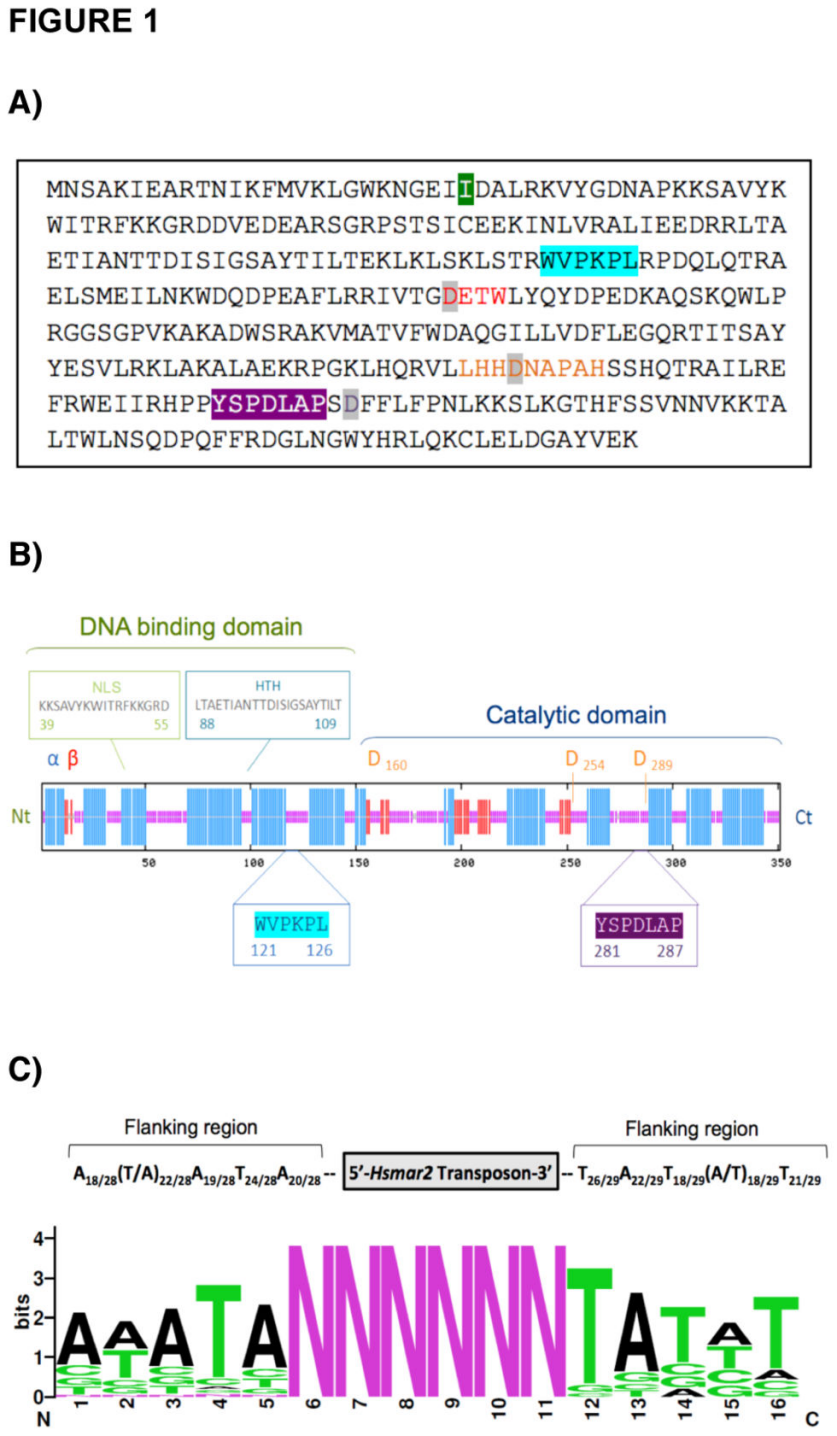
Characterization of *Hsmar2*. (**A**) Amino acid sequence of the *Hsmar2* transposase. The catalytic domain DD34D is highlighted in grey. The DETW and the LHHDNAPAH conserved motifs, which include respectively the first and second D residue of the catalytic domain, are in red and orange. The WVPKPL and the YSPDLAP motifs are highlighted respectively in blue and purple. The amino acid change compared to the consensus sequence U49974 is highlighted in green. (**B**) Predicted secondary structure of *Hsmar2*. The β-sheets are in red, and the α-helix in blue. The HTH domain predicted by NPS and the NLS domain predicted by PSORTII are indicated. (**C**) Values of the A/T prevalence of the 5 bp-long consensus sequences flanking the *Hsmar2* copies in the human genome and schematic representation using the weblogo program (http://weblogo.berkeley.edu/).

We have regenerated the mariner transposon *Hsmar2* by assembling and modifying sequences from inactive *Hsmar2* copies present in the human genome (Figure 1A). We detected the existence of many dinucleotides with similar frequencies for dinucleotide CG and dinucleotides CA or TG, and in all cases the hypermutable CpG dinucleotide was reintroduced in the sequence. Our sequence is very similar to the consensus sequence obtained by Robertson and colleagues [7] since the changes were introduced following the same criteria. However, there are six differences between the two sequences, but only one creates a change in an encoded amino acid: T to C at position 259, causing the change Thr_26_ to Ile_26_ (Figure S1A).

The *Hsmar2* transposon contains an open reading frame of 1053 bp, encoding a protein of 351 amino acids flanked by two inverted terminal repeated (ITR) sequences of 31 and 33 bp each, with an estimated molecular weight of 40.4 kDa. HSMAR2 contains the hallmarks of *mariner* transposase proteins, like the YSPDLAP motif and the catalytic triad DD34D [23]. The three aspartic acid residues are located at positions D _160_D_254_D_289_, and the first residue is part of the DETW motif conserved in the *irritans* subfamily. {Baba, 2005 #67}{Bell, 2007 #85} The secondary structure of the consensus HSMAR2 was predicted by Network Protein Sequence Analysis using the DSC [24] and PHD [25] methods and confirmed by the Predict Protein program [26] (Figure 1B). A putative HTH motif (helix-turn-helix), typically found in DNA transposases and involved in DNA binding, was predicted at position 88 but the score (0.58) was not significant. In addition, the program NUCDISC PSORTII [27] detected a bipartite Nuclear Localization Signal (NLS) at position 39, with a basic residue content of 16.5% and a score of 0.02. The ITRs size and non-identical sequences are characteristic of the *mariner* elements [28]. Nevertheless, due to our analysis, we introduced two changes in the ITR-3' compared to *Hsmar2* U49974: one T-insertion at position 1277 and a C to T substitution at position 1280 (Figure S1B). Moreover, we identified a 5 bp consensus sequence flanking *Hsmar2* elements, including the canonical TA dinucleotide (Figure 1C). We reasoned that these nucleotides may contribute to the recognition of ITRs in the transposition process and we therefore introduced them in the *Hsmar2* transposon.

Since transposons can be mobilized *in trans* by the transposases if they contain cognate ITRs, the HSMAR2 transposase gene was separated and replaced by other genes. In order to facilitate the characterization of HSMAR2 in cells in culture, a fusion protein between the C-terminus of GFP and HSMAR2, was generated and introduced into an adenovirus vector and Hela and HEK293 cells were subsequently infected. In addition, a polyclonal antibody (AbHsmar2-1001) against the N-terminal region of Hsmar2 was also generated and allowed the specific recognition and immunoprecipitation of the transposase (Figure S2A, S2B). Interestingly, the AbHsmar*2*-1001 antibody did not detect the transposase in non-transfected HeLa cells indicating that natural expression of HSMAR2 was null or very low in this cell line.

### Sub cellular localization of HSMAR2 and effect of its expression in the cell viability

**Figure 2:**
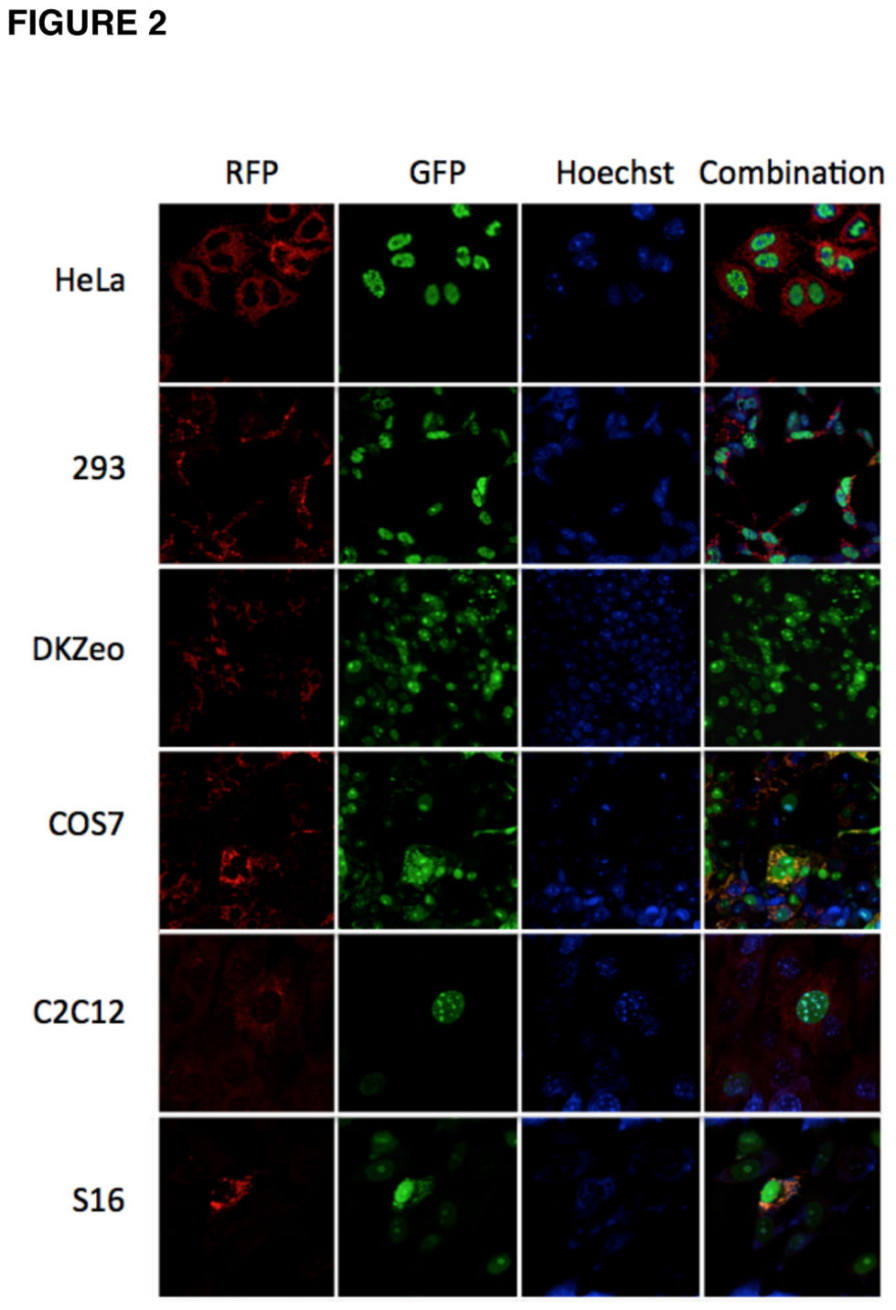
Subcellular localization of the HSMAR2. Different cell lines were infected (MOI = 30) with Ad/GFP-HSMAR2 and Ad/RFP. At 48h post-transfection cells were Hoechst stained and further analyzed by confocal microscopy.

To study the subcellular localization of the HSMAR2 transposase, different mammalian cell lines were infected with Ad/GPF-HSMAR2 and Ad5/RFP (as control). As observed in Figure 2 the fluorescence associated with the GFP-HSMAR2 fusion protein was mainly observed in the nucleus of cells, which is the expected localization for a protein that interacts with DNA.

**Figure 3:**
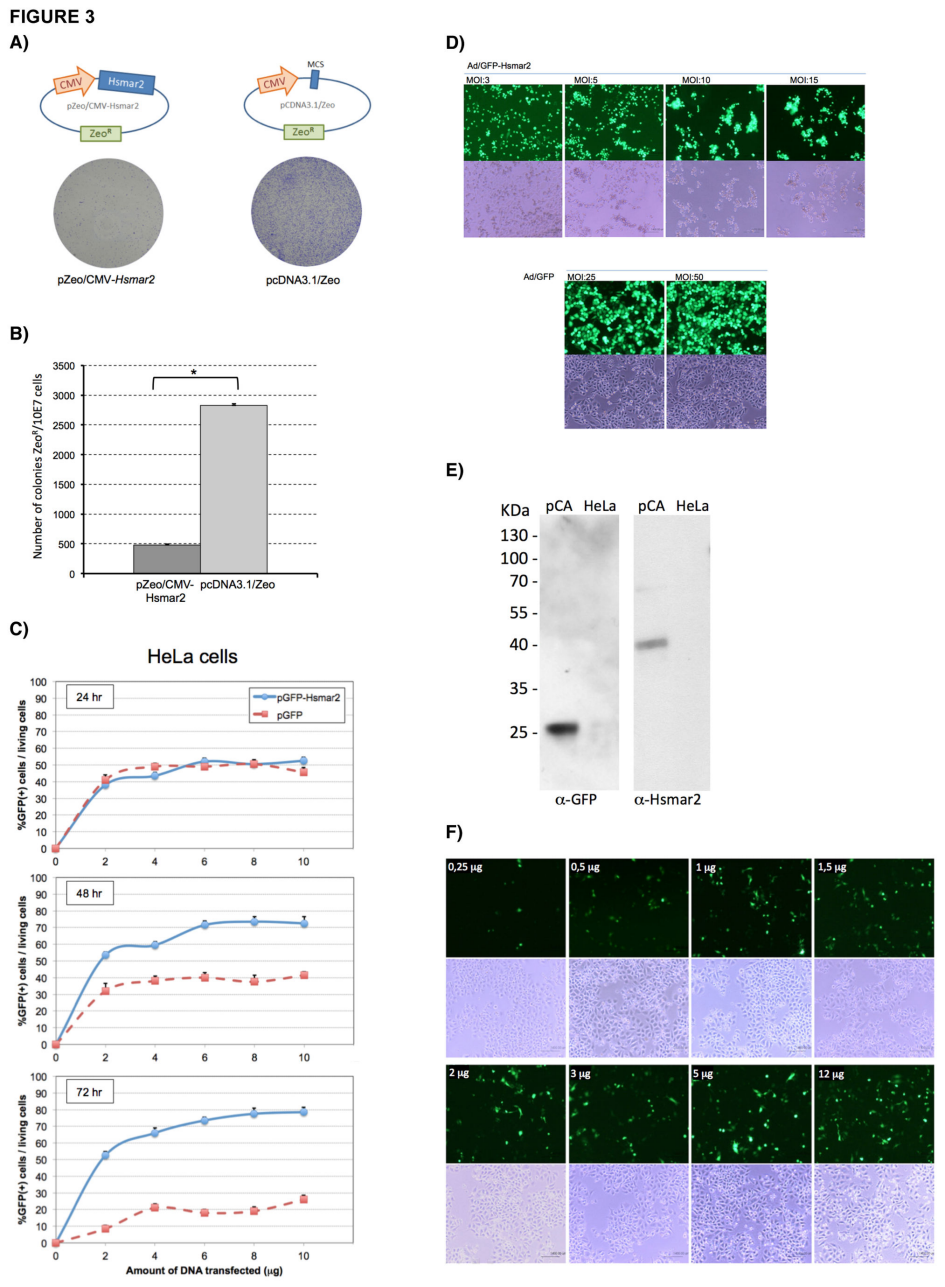
Overexpression of HSMAR2 **induces cytotoxity in HeLa cells**. (**A**) HeLa cells were transfected with pZeo/CMV-HSMAR2 and the control plasmid pcDNA3-Zeo and selected by Zeocin resistance for 15 days. Cell clones were counted after crystal violet staining. (**B**) Number of resistant Zeo-colonies per plate. The results come from two independent experiments. The asterisk indicates significant differences compared to control by Student’s t-test (p = 1,7E-07). (**C**) HSMAR2 induces cytotoxity at short time periods. The graphs represent the percentage of living cells (propidium iodide negative) expressing GFP- HSMAR2, for each dose of plasmid used. The results come from two independent experiments. (**D**) Images correspond to 72 hours post-infection of HeLa cells at increasing MOIs of Ad5/GFP-HSMAR2 (MOI 3, 5, 10 and 15) compared to infection of control Ad5/GFP (MOI 25 and 50). Each condition is visualized by both, fluorescence and bright field microscopy. (**E**) Western blot of protein extracts from HeLa cells transfected with the self-regulated expression plasmid pCA, using the AbHsmar2-1001 and anti-GFP antibodies. Same amount of total protein extracts were use in both conditions. Negative control: non-transfected HeLa cells. (**F**) Overexpression of the self-regulated HSMAR2 construct. The images correspond to the expression observed after 72 hours in HeLa cells transfected with increasing amounts of plasmid pCA (0.25, 0.5, 1, 1.5, 2, 3, 5, 12µg for 1.5 E + 06 cells).

In initial experiments, HeLa cells transduced with HSMAR2 showed changes in their morphology, had granules in the cytoplasm and detached from the plates preventing the progression of the experiment. To test whether overexpression of HSMAR2 was cytotoxic, we generated a plasmid carrying the Zeocin resistance gene (pZeo/CMV-HSMAR2) and the coding region of the HSMAR2 transposase. Transfected cells were selected for 15 days in order to measure the survival rates of HeLa cells expressing HSMAR2 by quantifying their capacity to generate Zeo-resistant colonies. As seen in Figure 3A and 3B, the number of Zeo-resistant colonies after transfection with the pZeo/CMV-HSMAR2 was 5-6 times lower than using a control plasmid without the HSMAR2 gene. To monitor whether it was possible to detect cytotoxicity due to overexpression of HSMAR2 early after transfection, HeLa cells were transfected with increasing doses of plasmid pGFP-HSMAR2 and analyzed by flow cytometry. As seen in Figure 3C, at 24 hours post-transfection the percentage of living cells expressing GFP is similar for both vectors. However, the percentage of living cells expressing pGFP-HSMAR2 decreased over time (45% to 26%), while transfection with the control GFP-plasmid increased the percentage from 52% to 79% (as GFP accumulated and was better detected over time), indicating that the cytotoxicity observed at 72 hours must be associated with the expression of HSMAR2.

To better study the mechanism involved in the HSMAR2-induced toxicity, HeLa cells were infected with increasing MOI of Ad5/GFP-HSMAR2. As expected, a gradual decline in the number of adherent cells and drastic changes in cell morphology were observed when increasing the amount of infecting adenovirus (Figure 3D). These adverse effects were seen even at low MOIs (MOI = 5). However, when infecting with the control Ad/GFP vector, cytotoxicity was not observed even at MOIs 10 times higher (MOI=50). Finally, to test whether there is any relationship between the nuclear localization of the HSMAR2 and cytotoxicity, we infected several mammalian cell lines (HeLa, DKZeo and COS7) with Ad/GFP-HSMAR2 at MOI=50 and analyzed cell viability 72 hours later. However, except in HeLa cells, there was no obvious toxicity (data not shown). Therefore, though nuclear localization of GFP-HSMAR2 may be required for cytotoxicity, it is not sufficient by itself, and other issues (i.e. the presence of specific host factors) may be involved in the process.

### Self-regulation of HSMAR2 expression

Due to the cytotoxicity caused by HSMAR2 overexpression over time, we generated a negative self-regulated expression cassette, where the HSMAR2 expression decreases as the effect of its own transposase action, thus avoiding the prolonged expression of HSMAR2. To this end, we constructed the plasmid pCA, where the ITR sequences flanked both, a GFP expression cassette, as well as the CMV promoter driving the expression of HSMAR2, while the transposase gene was inserted outside of the ITRs. HeLa cells were transfected with pCA and 48 hours post-transfection, both the GFP gene within the transposon, and the HSMAR2 transposase were efficiently expressed (Figure 3E). Interestingly, when HeLa cells were transfected with increasing amounts of plasmid, cell viability was similar in all conditions even when high levels pCA were used (Figure 3F) indicating that the self-regulated expression of HSMAR2 prevents the cytotoxic effects associated with its continuous expression.

### HSMAR2 is able to bind specifically its own ITRs

**Figure 4:**
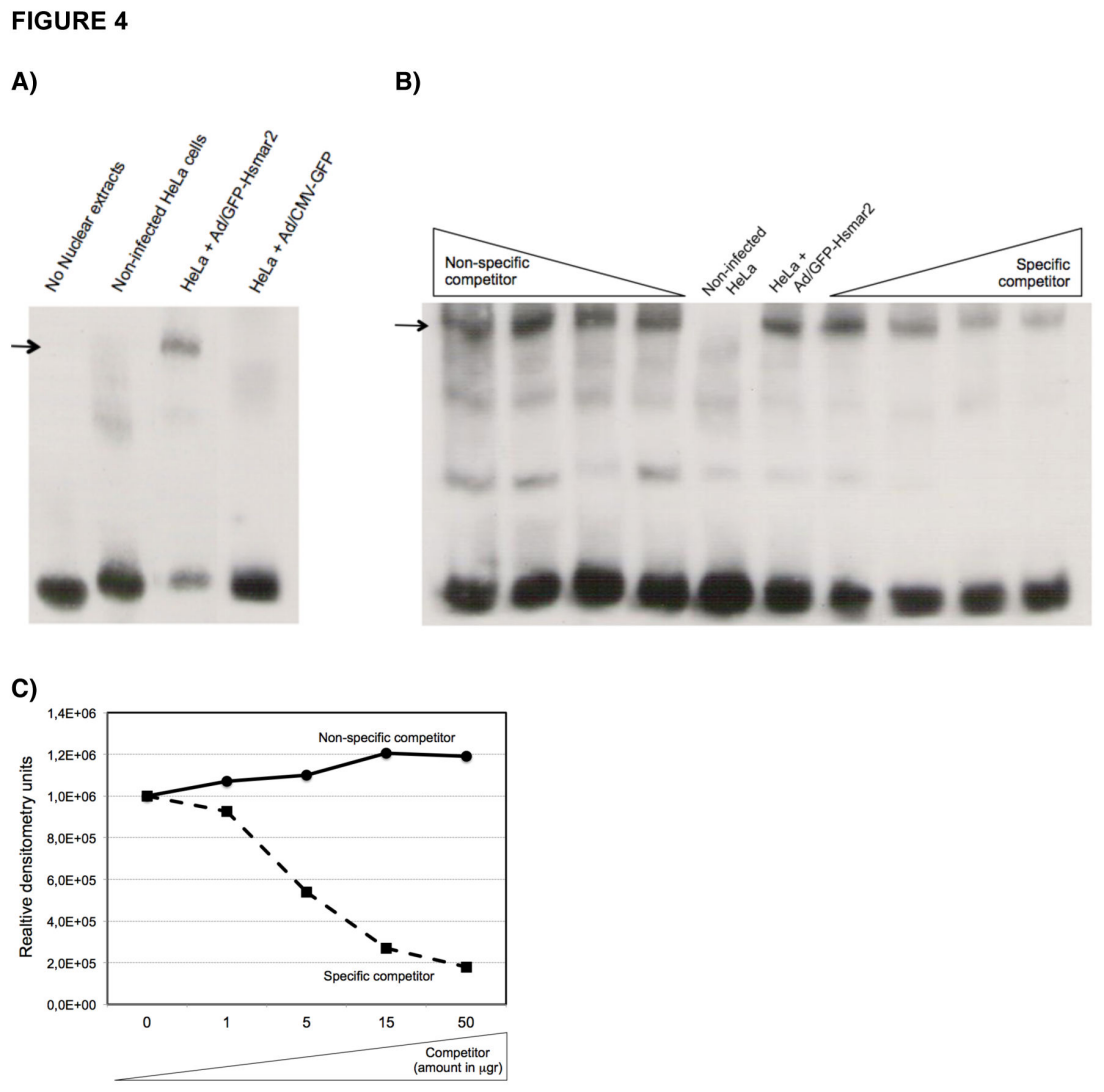
Binding of the HSMAR2 **transposase to its ITR sequences**. (**A**) EMSA assay. Detection of the binding of Hsmar2 to the ITR sequence. Double-stranded oligonucleotides with the ITR sequence biotin-labeled were incubated with nuclear extracts from HeLa cells infected with adenovirus Ad/EGFP-HSMAR2 (third lane). The retarded band is marked with an arrow. Controls are: no incubation with nuclear extracts (first lane), with extracts from non-infected HeLa cells (second lane), and incubation with HeLa cells infected with an adenovirus not expressing HSMAR2. (**B**) Competitive EMSA using nuclear extracts of HeLa cells infected with Ad/GFP-HSMAR2. The major retarded band is marked with an arrow. Increasing amounts (1, 5, 15 and 50 nM) of a biotin-labeled non-specific competitor (first four lanes) or of unlabeled duplex oligonucleotides of the 5’-ITR of *Hsmar2* (last four lanes) were used. The sixth lane shows the result of the binding reaction using no competitor. (**C**) Graph based on the intensity of the bands delayed, from (B). Relative densitometry units refers to the direct quantification of the band signal by densitometry.

The first step of the transposition reaction is the binding of the transposase to the terminal ends of the transposon. To determine whether HSMAR2 is able to recognize and bind its own ITRs specifically, HeLa cells were infected with Ad/GFP-HSMAR2 and analyzed by mobility shift assay (EMSA) using oligonucleotides from the 5’-ITR labeled with biotin. As seen in Figure 4A, a retarded band is detected only when nuclear extracts were obtained from cells infected with the Ad/GFP-HSMAR2 vector. The retarded band was not observed in any of the controls, including in HeLa cells infected with Ad/CMV-GFP. Next, competitive EMSAs were performed to determine the level of specificity of the interaction between HSMAR2 and ITR, and designed following a double strategy: competition with non-labelled ITR-oligonucleotides, and competition with randomized ITR-oligonucleotides (Figure 4B). Interestingly, increasing amounts of the specific competitor gradually inhibited the binding of HSMAR2 to the ITR sequence up to more than 80%. However, the binding of HSMAR2 to the ITR was not altered by the presence or excess of non-specific competitor with a randomized sequence. Therefore, HSMAR2 is capable of forming nucleoproteic complexes with its own ITRs and this interaction is sequence specific. Finally, to analyze whether the HSMAR2 transposase is also able to recognize other transposons of the Tc1/mariner superfamily as for example Sleeping Beauty, HeLa cells were cotransfected with the HSMAR2 transposase plus the the *Sleeping Beauty* transposon (pIR-neo), which contains their specific inverted repeats (IR) flanking the neomycin resistance gene; or with the Sleeping Beauty transposase together with the HSMAR2 transposon pITR (CG+5T)-neo. As expected, no cross-recognition between the two transposases was observed (Figure S3).

### HSMAR2 excision and footprint analysis

**Figure 5:**
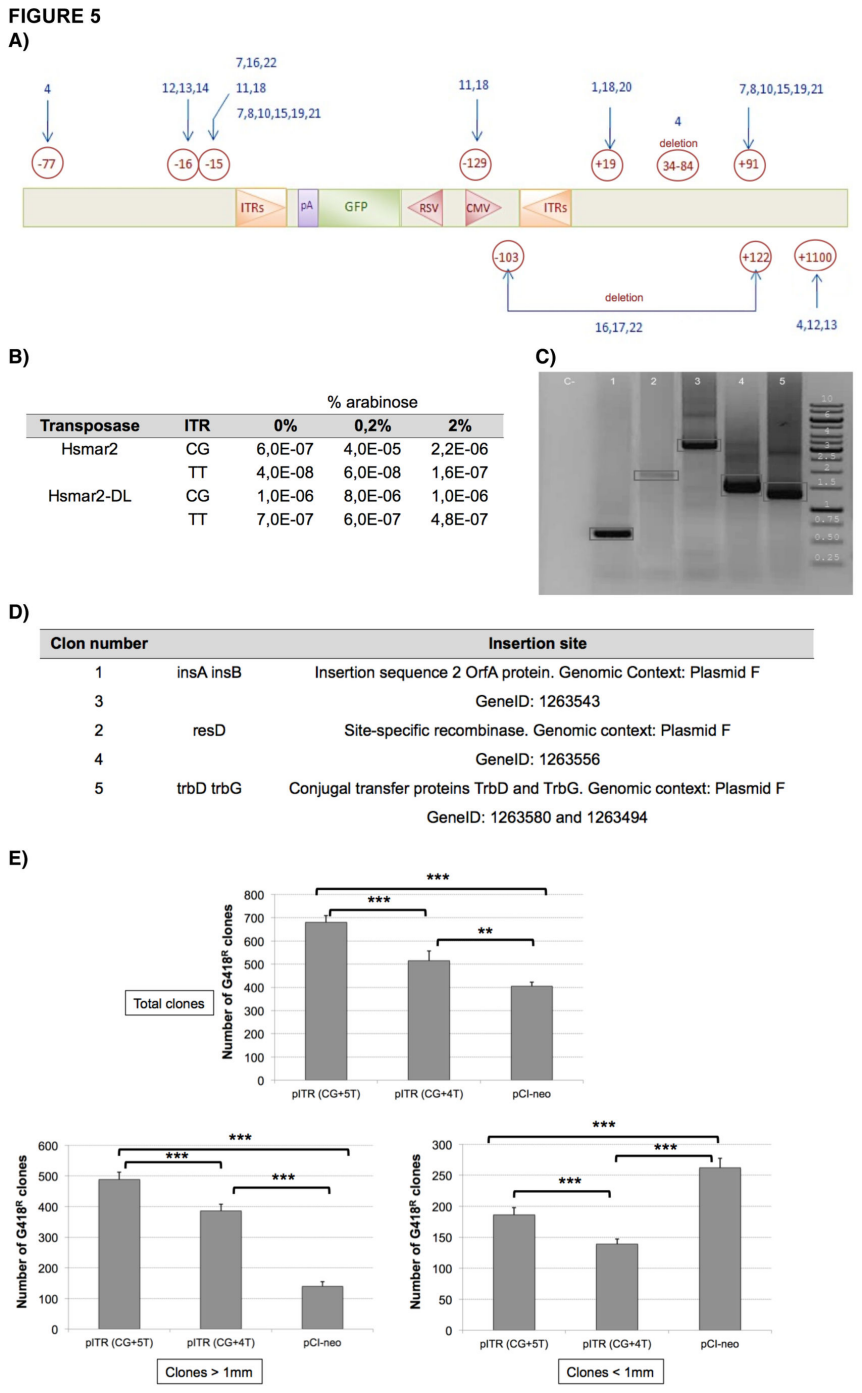
Analysis of the excision and transposition processes. (**A**) Excision sites for the HSMAR2 transposase. Non-circled numbers correspond to the clone numbers, while encircled numbers correspond to the cleavage sites. (**B**) Transposition frequencies obtained with the mating-out assay (**C**) Amplification of the regions flanking the integration points. Gel bands (boxed) were cut and sequenced. DNA samples from the F plasmid from independent colonies of the mating-out (NalR + KanR + GentR) assays. 1: TT-clone, 2: TT-clone, 3: CG-clone, 4: CG-clone, 5: CG-clone. (**D**) Localization of the integration sites of the transposon ITR-Kan^R^ into the target plasmid pOX38. It shows the site of transposon insertion by analyzing the sequence obtained by BLAST. (**E**) Number of G418-resistant colonies after transposition in HeLa cells. The values correspond to the average of the neomycin-resistant clones of four independent experiments with n=3 per condition. It also shows the same results into two separate graphs according to whether the diameter of the cell clone if less than or equal to 1mm or is greater than 1mm. The asterisks indicate significant differences (the Student’s t-test, *p < 0,01; **p<0,001). Negative control (pCIneo) corresponds to a cotransfection of the Hsmar2 transposase with a plasmid carrying the Neo cassette but without the ITRs. In all cases, transfection conditions were 6 µg of plasmid per million cells.

To study the excision step we analyzed the donor molecule because after transposition and the subsequent DNA repair process, the donor plasmid is smaller and can be detected with external primers flanking the ITRs. To this end, HeLa cells were transfected with the self-regulated pCA plasmid. After 72 hours of transfection, low molecular DNA was extracted by the Hirt method and digested by restriction enzymes cutting within the transposon, and finally PCR amplified, cloned and sequenced. If transposon excision was accurate and canonical, all sequences should be identical. However, the HSMAR2 transposase did not excise the transposon perfectly, but utilized cutting sites before and after the ITRs (Figure 5A). Thus, for the 5’-ITR, two major excision sites were detected at positions -15 and -16, while for the 3’-ITR the cutting reaction showed more variability, with deletions and even cuts inside the expression cassette between the ITRs, suggesting that though the regenerated HSMAR2 transposase has nicking activity, its catalitic domain must not be fully functional.

### Analysis of the transposition process in prokaryotes mediated by HSMAR2

The transposition assay for conjugation (mating-out assay) measures in bacteria the frequency of transposition of a Kanamycin resistance gene, flanked by ITRs, to an F plasmid. Based on previous experiments in *Himar1* by one of us (DJL) [29], where overexpression of HIMAR1 is associated to self-inhibition, the HSMAR2 motif WVPKPL was mutagenized to the canonical motif WVPREL to thus generate HSMAR2-DL, and therefore facilitate the generation of hyperactive constructs. In addition, since overexpression of HIMAR1 is also associated with cytotoxicity [30], we cloned, both the coding sequence for the consensus HSMAR2 and the mutagenized HSMAR2-DL under the control of the arabinose inducible ara_BAD_ promoter (Figure S4).

In order to check whether HSMAR2 has also a preference for certain sequences at the ends of the ITRs, we also generated two constructs carrying a kanamycin resistance gene, where the flanking ITRs show slight differences in their sequences, as the outer two nucleotides were changed from CG to TT. As seen in Figure 5B both, HSMAR2 transposases (HSMAR2 and HSMAR2-DL) are able to transpose in *E. coli* at similar levels, suggesting that the WVPKPL motif of HSMAR2 is probably under natural selection to produce inhibition by overexpression, which it would open the door to generate hyperactive constructs of HSMAR2. We also observed better efficiency when the transposon contained the CG dinucleotide at the end, instead of the TT dinucleotide. To analyze the integration sites to the target plasmid, DNA of the F plasmid was extracted from independent colonies, digested with restriction enzymes, recirculated, analyzed by inverted PCR followed by a nested PCR (Figure 5C), and sequenced. Interestingly, we detected several independent events within the same gene in more than one sample, indicating that, at least in the target plasmid pOX38, HSMAR2 shows preference for some regions (Figure 5D). However, as it happened for the excision process, no events involving the TA dinucleotide were observed.

### Analysis of the transposition process in eukaryotes mediated by HSMAR2

HeLa cells were cotransfected with the pITR-neo and pGFP-HSMAR2 plasmids system and grown in selective conditions in the presence of G418 for at least two weeks, until individual clones appeared. To evaluate the non-specific integration, a control plasmid (pCI-neo) without the ITR sequences was used. Moreover, we also used a new ITR-construct carrying one thymidine less in the 3’-ITR sequence (construct CG+4T) to mimic the 3’-ITR of the consensus U49974 sequence of *Hsmar2* (Table S1) and thus study the effect of this sequence variation since the *Hsmar2* sequence has 5T within the 3’-ITR (CG+5T). As seen in Figure 5E a significantly higher number of neomycin-resistant clones is observed when both, the HSMAR2 transposase and the transposon are co-transfected. We also observed that HSMAR2 seems to show preference for the ITRs with the CG+5T sequence, as the number of clones is higher when the cells are transfected with this transposon. These results suggest that the HSMAR2 protein may have transposase activity, and second, that the transposon ITR-neo contains *in cis* the required sequences for transposition, with an activity activity similar to that of other elements as *SB11*, *piggybac* or *Tol2* in HeLa cells [31]. Some authors have hypothesized that the size of the clones may be used as an indication of the transposition, because after 2 weeks of selection, colonies smaller than 1 mm are difficult to expand in the presence of the antibiotic and are considered to be non-specific clones or to have unstable inserts [32]. In this regard, when only colonies bigger than 1 mm are taken into account, the transposition efficiency is significantly higher (2-3 times) in plasmids containing ITR-transposons than in control plasmid (p=0,0003 for pITR CG+5T; p=0,001 for pITR CG+4T), while when taking into account smaller colonies, the profiles are inverted and the efficiency is reduced by 30% to 50% (Figure 5E).

## Discussion

In order to increase the applications of Tc1/*mariner* transposons as genetic tools it is desirable to obtain and characterize new transposables elements with different properties. To this end, we have regenerated the human *mariner* transposase HSMAR2 and characterized its activity, and have observed toxicity by overexpression, which led us to generate a system for the self-regulated expression of the transposase. The transposition process of DNA fragments follows a cut and paste mechanism consisting of several steps that include binding of the transposase sequences to the terminal ITRs to form a synaptic complex, the excision of the transposon from the donor molecule, and the integration into a new site in the genome. For the HSMAR2 transposase we have been able to detect and characterize each of the successive steps of the transposition process: the capacity of HSMAR2 to recognize and bind specifically to its terminal repeat sequences, as well as the excision of the transposon, documented by the footprints left in the donor molecule.

After regenerating a copy of the human mariner HSMAR2 we demonstrated that it can be stably expressed in human cells, and that we were able to detect it by generating specific antibodies. Moreover, the fluorescence associated with the expression of GFP-HSMAR2 was localized primarily into the nucleus of HeLa cells, which is the expected localization for a protein that interacts with DNA and suggests that HSMAR2 must be actively transported to the nucleus and therefore it must have a functional NLS in its sequence. In fact, since proteins of more than 60 kDa require selective transport mechanisms to pass through the nuclear pore complex [33] GFP-HSMAR2 (molecular weight of about 70 kDa) must be actively imported into the nucleus after being synthesized in the cytoplasm, suggesting that it should have a functional NLS in its sequence. In this regard, most of the active transposases have nuclear localization signals. For example, the SETMAR transposase, which has a partial transposase activity, is localized in the nucleus of HEK293 cells and the nuclear extracts can be used in in vitro assays [34]. However, in the absence of structural information on HSMAR2, we can only infer the presence of certain structural motifs. Thus, the predicted secondary structure of HSMAR2 by some bioinformatics programs suggests the presence of a bipartite NLS, though it does not seem to be a classic NLS signal. However, other programs did not detect any NLS sequence and in fact, the majority of programs identified HSMAR2 as a cytoplasmic protein.

Interestingly, overexpression of HSMAR2 in HeLa cells is cytotoxic. What it governs this effect is not known, although it may be associated with its nuclear localization. Thus, it is likely that due to the existence of inactive copies of the transposase HSMAR2 the human genome, its overexpression in human cells may cause either the generation of nicks into the DNA or uncontrolled movements of these inactive copies, triggering the activation of cellular apoptotic pathways in a process similar to that observed in the overexpression of other proteins with binding and cutting capacity of DNA molecules, such as the Cre recombinase, which is associated with cell toxicity probably due to the existence of cryptic binding sites for the recombinase or pseudo loxP present at the genomes of mammals [35]. Despite these results, localization into the nucleus is not sufficient to cause cytotoxicity and other issues (i.e. the presence of specific host factors) might have a role.

The ability of *mariner* elements to act with relative independence of species-specific factors is exemplified by the diverse range of hosts, and could help explain their ability to transfer horizontally to new hosts. For example, the *Sleeping Beauty* transposase is active in most mammalian cells but the frequency of transposition varies between cell lines as well as in vivo, being higher in murine germ cells that in murine embryonic stem cells, suggesting the existence of mechanisms regulating their transposition. However, it cannot be excluded that factors encoded by the host cell in some organisms are necessary for transposition in vivo because the absence of these factors could explain why transposition is not always detected and why it has so much variability [31,36]. Moreover, it has been reported that *Himar1* shows differences in the frequency of transposition between different clones of HeLa cells, which could be due to differences between levels of a cellular factor that modulates the efficiency of transposase [29].

As expected, the HSMAR2 transposase binds specifically to its terminal sequences. In addition, there is no cross-recognition between the *Sleeping Beauty* and HSMAR2 transposases. Consistently, it has been reported that the MAR region of the *Hsmar1*-derived element SETMAR does not recognize the ITR-*Hsmar2* sequences [37]. After the transposase: ITR interaction, the double strand breaks generated during the excision of the transposon are likely repaired by a mechanism of non-homologous end joining (NHEJ) [36,38]. The double strand breaks stimulate repair processes in mammals by homology directed repair mechanims, and so it has been proposed that non-canonical footprints could be due to disruption of these processes, particularly for interruptions in the repair mechanisms of the homologous recombination of double-stranded DNA cuts which, together with binding processes of the DNA ends, would result in microhomologies [39]. In this regard, after the excision process, although some canonical events produced by NHEJ are expected, the footprints left by HSMAR2 were not canonical, especially at the 3’-ITR where more variability was observed. Interestingly, similar results have been described for other transposases, with footprints of different sizes including non-canonical footprints like gaps and insertions. For example, the SETMAR protein is only capable of cutting the 5'-end, by generating footprints associated with small extensions, deletions and micro-homologies at the excision sites [40]. Similarly, the *Sleeping Beauty* transposase showed variable results depending on the cell type used: mostly canonical footprints obtained in zebrafish, and hepatocytes and embryonic stem cells from mice [41,42], while non-canonical footprints are found in HeLa cells in culture [41] and in haploid espermatids in mouse [36]. However, despite the ability of the transposase itself to perform cut and paste transposition from TA parental sites to TA target sites, this phenomenon likely reflects the different availability of NHEJ repair factors in different cell lines either because the host repairs the excision sites with different efficiency, and/or homologue-dependent repair pathways operate more dominantly in certain cells than NHEJ.

Until now, the strategies to generate improved versions of tranposases have been based primarily on random mutagenesis with genetic selection and subsequent functional analysis of each mutated version. Thus, the *Himar1* transposase was mutagenized and hyperactive constructs selected, such as the C9 version which is currently used as a genetic tool [30]. This work also studied the effects of dominant negative inhibition of mutated variants of *Himar1*, showing that the highly conserved domain WVP(R/H) EL of *mariner* elements generated hyperactive mutants in papillation assays when the W, V, P and E amino acids were mutated to alanine [43]. Moreover, in the *Mos1* element this motif is involved in selecting the integration site, since mutations in this site permit a relaxation of the specificity, including integrations also in the dinucleotide CG [44]. Interestingly, despite HSMAR2 does not have the consensus WVP(R/H) EL motif, the values obtained in the mating-out assay are similar to the HSMAR2 mutant construct carrying it (HSMAR2-DL) suggesting that the WVPKPL motif of HSMAR2 is probably under natural selection to produce inhibition by overexpression, which it would open the door to generate hyperactive constructs of HSMAR2. In addition, the frequency was clearly higher when the transposon was flanked by the ITRs ending with the canonical GC dinucleotide. More important, the values were similar to those found for IS elements (of the itm superfamily) [45] and Tn*5* and Tn*7* elements [46,47], or for *Himar1* in similar mating-out assays [30]. However, although HSMAR2 can be transported to the nucleus, can specifically bind to Hsmar2-ITRs *in vitro* (thus the DNA-binding helices and the NLS of the transposase version are functional), and transposes Hsmar2-ITR flanked sequences preferently to specific target regions, neither the excision nor the transposition processes were canonical in both bacteria and human cells, and therefore, despite the catalytic domain has nicking activity is not able to cut and paste the transposon precisely at the TA dinucleotides. Due to it, future studies to reconstruct and optimize the catalytic domain of HSMAR2 must be addressed.

In summary, we observe that the sub-cellular localization of HSMAR2 is dependent on the host cell type and that HSMAR2 is cytotoxic when overexpressed in HeLa cells. T herefore the use of self-regulated cassette makes the HSMAR2 an interesting tool for genetic studies, while the development of specific antibodies may facilitate the study of the *Hsmar2* association to hotspots for homologous recombination involved in human genetic disorders as Charcot–Marie–Tooth, Prader-Willi, Angelman, and Williams syndromes.

## Supporting Information

Figure S1 **Sequence of the transposable element *Hsmar2* reconstructed by our group.** (**A**) Sequence of Hsmar2. ITRs are shown in green, with the sequence 5'-CGAGGG-3' marked in yellow. In black are non-translated regions (UTR) underlined, and in the blue region coding for the transposase protein (CDS, coding sequence), with START and STOP codons highlighted in capitals. In orange are the changes compared with the consensus sequence published by Robertson (GenBank Accession No. U49974, Protein ID AAC52011.1 UniProt entry Q13539) (Robertson HM, Martos R, 1997, Gene 205: 219-228). (**B**) Two changes were introduced in the ITR-3' compared with *Hsmar2* U49974: one T-insertion at position 1277 and a C to T substitution at position 1280.
(TIF)

Figure S2 **(A) Western blot using specific antibodies against Hsmar2 and against GFP.** HeLa cells were transfected with pGFP-Hsmar2, pKS-RSV/GFP or non-transfected. (**B**) Specific Immunoprecipitation of Hsmar2 and western blotted using an antibody against GFP.
(TIF)

Figure S3 **Absence of cross-recognition between the transposases *Hsmar2* and Sleeping Beauty (SB).** Cotransfection in HeLa cells with the indicated combinations of plasmids and subsequent selection for two weeks in the presence of G-418. Cell clones were visualized after crystal violet staining.
(TIF)

Figure S4 **Analysis of the excision and transposition processes.** Detection of inducible Hsmar2 expression in *E. coli* with increasing concentrations of arabinose (0 to 0.2%). Protein extracts from bacterial cultures, previously induced with increasing concentrations of arabinose, showed a band of the expected size of Hsmar2 in denaturing polyacrylamide gels Detection with antibody specific AbHsmar2-1001.
(TIF)

Table S1 **Sequences of the ITRs Hsmar2 used in the transposition assay in HeLa cells.** CG or TT dinucleotides are in bold, and the 4T or 5T sequences are underlined.
(TIF)
