# Supplementary figures and images for: Functional Characterization of the Human Mariner Transposon Hsmar2
Source: PLoS One. 2013 Sep 11;8(9):e73227. doi: 10.1371/journal.pone.0073227 (PMC3770610; doi:10.1371/journal.pone.0073227)

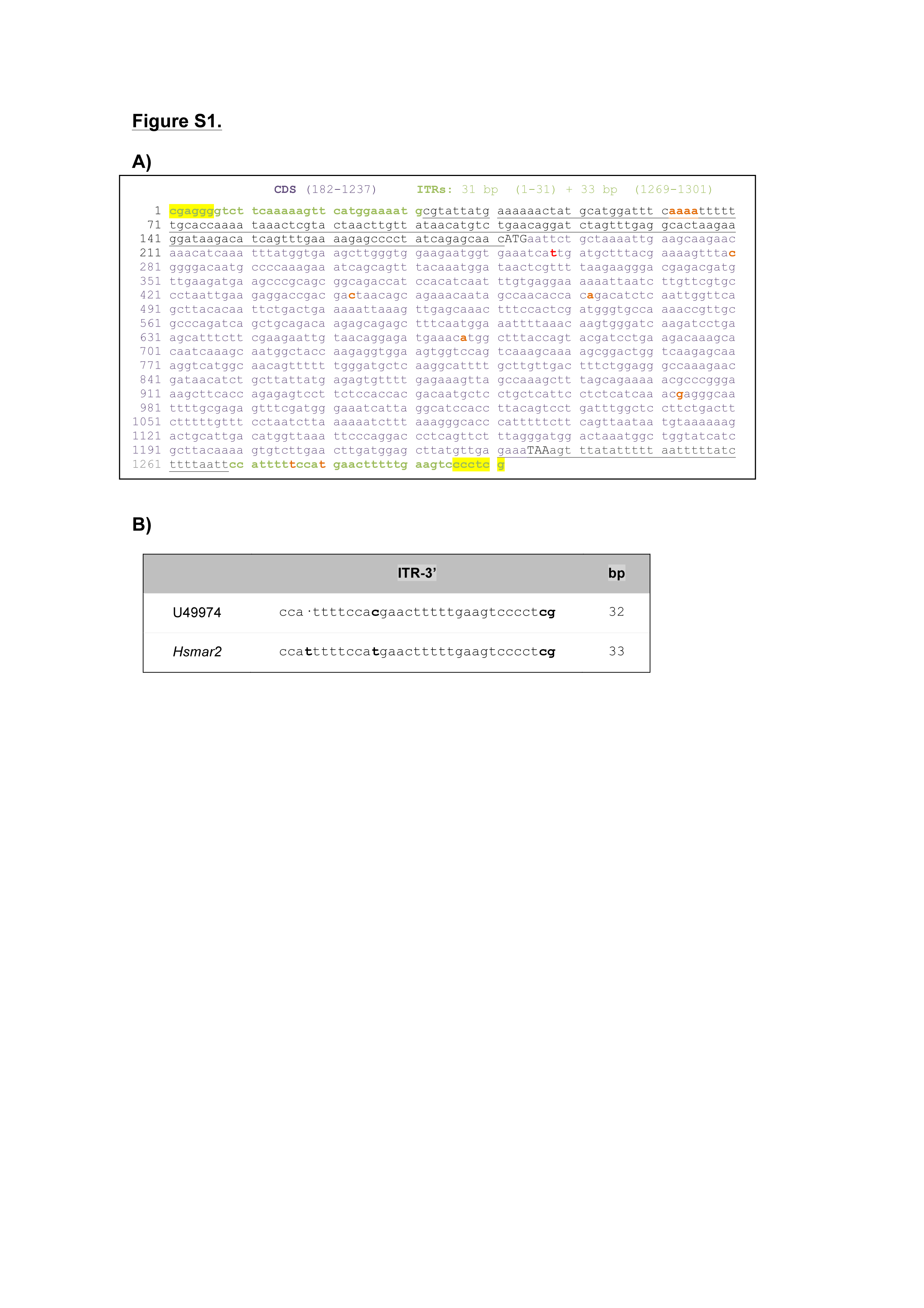

Supplement: Figure S1 — Sequence of the transposable element Hsmar2 reconstructed by our group. (A) Sequence of Hsmar2. ITRs are shown in green, with the sequence 5'-CGAGGG-3' marked in yellow. In black are non-translated regions (UTR) underlined, and in the blue region coding for the transposase protein (CDS, coding sequence), with START and STOP codons highlighted in capitals. In orange are the changes compared with the consensus sequence published by Robertson (GenBank Accession No. U49974, Protein ID AAC52011.1 UniProt entry Q13539) (Robertson HM, Martos R, 1997, Gene 205: 219-228). (B) Two changes were introduced in the ITR-3' compared with Hsmar2 U49974: one T-insertion at position 1277 and a C to T substitution at position 1280. (TIF) [file pone.0073227.s001.tif]

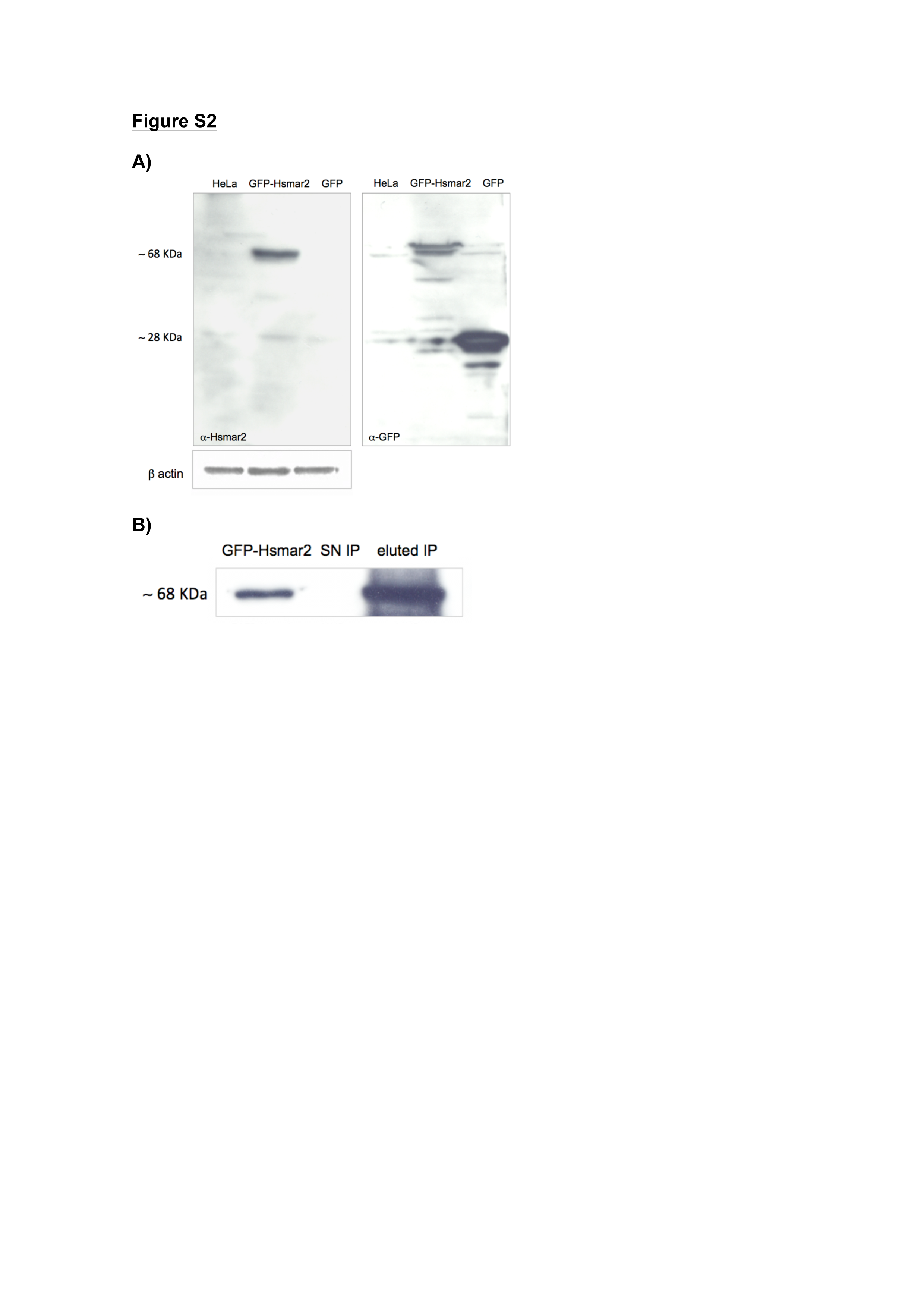

Supplement: Figure S2 — (A) Western blot using specific antibodies against Hsmar2 and against GFP. HeLa cells were transfected with pGFP-Hsmar2, pKS-RSV/GFP or non-transfected. (B) Specific Immunoprecipitation of Hsmar2 and western blotted using an antibody against GFP. (TIF) [file pone.0073227.s002.tif]

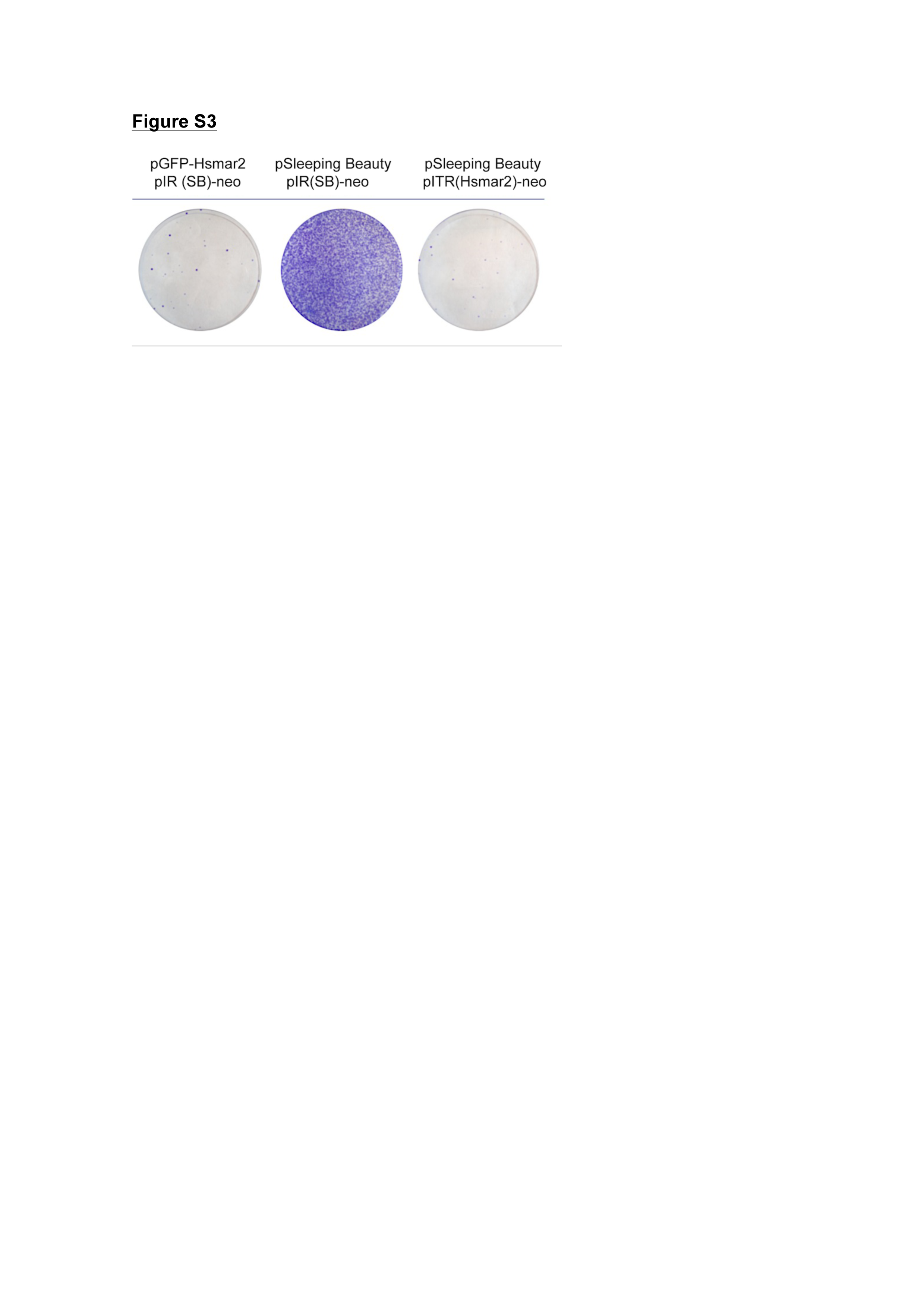

Supplement: Figure S3 — Absence of cross-recognition between the transposases Hsmar2 and Sleeping Beauty (SB). Cotransfection in HeLa cells with the indicated combinations of plasmids and subsequent selection for two weeks in the presence of G-418. Cell clones were visualized after crystal violet staining. (TIF) [file pone.0073227.s003.tif]

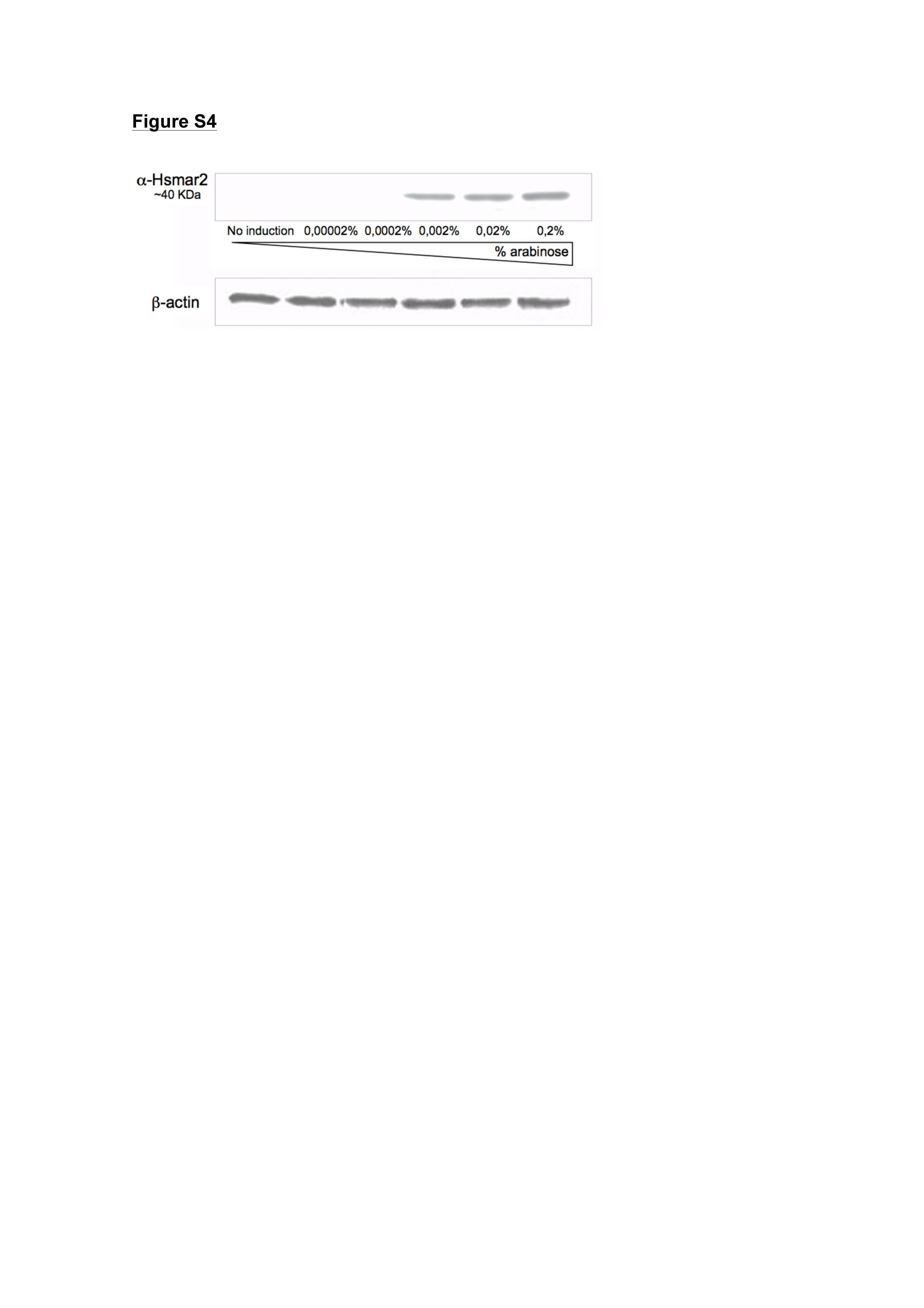

Supplement: Figure S4 — Analysis of the excision and transposition processes. Detection of inducible Hsmar2 expression in E. coli with increasing concentrations of arabinose (0 to 0.2%). Protein extracts from bacterial cultures, previously induced with increasing concentrations of arabinose, showed a band of the expected size of Hsmar2 in denaturing polyacrylamide gels Detection with antibody specific AbHsmar2-1001. (TIF) [file pone.0073227.s004.tif]

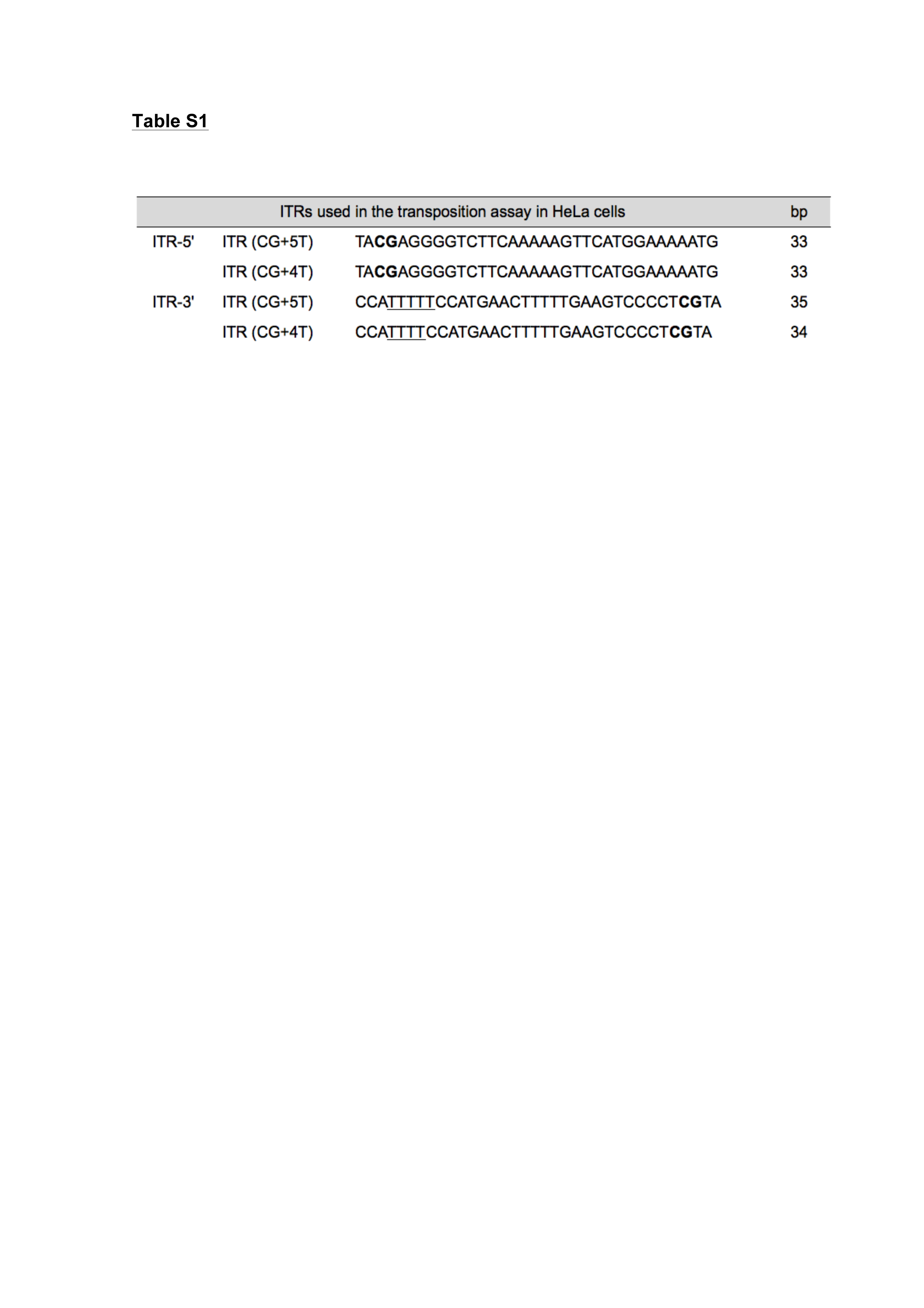

Supplement: Table S1 — Sequences of the ITRs Hsmar2 used in the transposition assay in HeLa cells. CG or TT dinucleotides are in bold, and the 4T or 5T sequences are underlined. (TIF) [file pone.0073227.s005.tif]
